# Hormonal and neuromuscular responses during a singles match in male professional tennis players

**DOI:** 10.1371/journal.pone.0195242

**Published:** 2018-04-06

**Authors:** Álvaro López-Samanes, Jesús G. Pallarés, Alberto Pérez-López, Ricardo Mora-Rodríguez, Juan F. Ortega

**Affiliations:** 1 Exercise Physiology Laboratory at Toledo, University of Castilla-La Mancha, Toledo, Spain; 2 School of Physiotherapy, Faculty of Health Sciences, Francisco de Vitoria University, Madrid, Spain; 3 Faculty of Sports Sciences, University of Murcia, Murcia, Spain; 4 Departament of Medicine and Medical Specialties and Department of Biomedical Sciences. Faculty of Medicine and Health Science, University of Alcala, Madrid, Spain; Universidad Europea de Madrid, SPAIN

## Abstract

We sought to measure the response of cortisol concentrations around a professional tennis match and its association with hydration status and neuromuscular performance. Nine professional male tennis players were tested in a rest day, and 2-week after, during the first match of a professional tournament played in a clay-court. Salivary concentrations of cortisol (SalCC) were measured in a resting day (9:00 am and 8:00 pm), at the match day (9:00 am and 8:00 pm) and immediately before and after the match. Hydration status was assessed before the match (urine specific gravity; USG) while fluid turnover was tracked during the match. Finally, counter movement jump (CMJ) and handgrip isometric strength (HS) were measured before and after the match. SalCC, either in the morning (P = 0.161) and afternoon (P = 0.683) was similar in rest and match days. However, SalCC increased after the match (P = 0.033). Participants started the match hypohydrated (USG = 1.026±0.002) and during the match lost 1.0±0.3% of body weight despite 1.035±0.124 L/h of fluid ingested. CMJ and HS did not change post-match (P = 0.210 and P = 0.881, respectively). Correlations between the elevations in SalCC and dehydration (% BW loss) during the match were significant (r = -0.632; P = 0.034). Professional male tennis players did not show an anticipatory increase in SalCC the day of the match and neither signs of neuromuscular fatigue after it. During the match, the mild dehydration (i.e., <1.5%) was associated with the increases in cortisol levels which suggests that dehydration may be an added stress to be considered.

## Introduction

Tennis is an intermittent sport played around the world by more than 83 millions of people, including over 4000 elite junior tennis players currently ranked by the nternational Tennis Federation[1] During a tennis match, players execute high-intensity actions characterized by short bouts of high intensity exercise (4–10 seconds), which are composed by accelerations/decelerations, strokes and change-of-direction (COD) interspersed with periods of low-moderate intensity or rest (short break between points (10–20 seconds) and moderate rest between games and sets (90–120 seconds), with match duration between 1–5 hours[2, 3]. Demands of a tennis match may produce decrease in neuromuscular performance as a consequence of alterations in the mechanisms involved in muscle activation. Decrease in neuromuscular performance has been studied in several exercise/sports modalities using a relatively simple and feasible test, the counter-movement jump (CMJ)[4]. When neuromuscular performance decreases, it is denominated as neuromuscular fatigue (NMF). Research conducted during tennis national tournaments [5] and simulated tennis matches [6] have identified NMF by decrements either in the rate of force development [5] or the maximal voluntary contraction[6]. Moreover, the response of stress hormones like cortisol has been shown to be significantly increased during a tennis match[7, 8].

Professional tennis tournaments (i.e. ATP, Challengers and Futures) are scheduled around the world with a tight calendar which implies competition almost every week of the year. Hence, for professional players the physiological challenge of prolonged and frequent trips adds to the neuromuscular and physiological demands during the competition[9]. To minimize and control the incidence of these demands and to ensure adequate recovery between matches and tournaments tennis players are commonly advised to use nutritional supplements during the season [10, 11] as well as to adequate their behavioral/chronobiological patterns (e.g. sleep-hygiene, training at the schedule of competitions) and recovery strategies (cold-water immersion, compression garments) to maximize performance)[9, 12].

Several methods of assessing exercise-related physiological stress have been proposed, being the determination of the circulating levels of hormones (i.e., testosterone and cortisol concentrations [13] and the use of psychological questionnaires (e.g., CSAI-2R[14]) the most frequently used. Therefore, serum cortisol concentration has been used as physiological stress marker in individual[15] and team sports (e.g., soccer)[16]. Historically, cortisol concentrations have been determined from blood samples, however, the determination of cortisol in other body fluids, such as saliva, have been recently adopted since it is a non-invasive technique[17], and the results are highly correlated to serum cortisol concentration (r = 0.620, P<0.001;[18]). Thus, cortisol concentrations measured in blood or saliva are valid methods to measure physiological stress in tennis. Nonetheless, the effects of a tennis match in cortisol levels have only been described in amateur players but, to our knowledge, not in professional male tennis players competing in a professional tennis tournament (i.e., Futures).

It has been reported that extreme elevations of serum cortisol concentration may interfere with decision making which could in turn affect performance[19]. In amateur tennis players, Filaire et al., [8] found an anticipatory increase in SalCC the morning before a match of a regional tournament. In addition, the authors determined that those players who lost their match showed higher cortisol concentrations prior to the game in comparison to those who won their match. However, to our knowledge, similar findings as those described by Filaire et al., study [8] have not been conducted during professional tournaments where players are more experienced and money prizes are involved.

NMF has been assessed in tennis using a variety of test such as, jump heights, sprint running, serve velocity and agility, since those have been regarded as key determinants of tennis performance[20–22]. It has been demostrated in young tennis players that two [23] and four-days of consecutive matches results in NMF[24]. However, there are scarce evidences about the identification of NMF after a single match in professional tournaments. Pereira and co-workers analysed physical performance in profesional players during a single match and they did not find any significant difference in lateral and forward displacement, distance covered per point, game and set, and the rate of time spent in each range of velocity, between the second and the first set[25]. The authors highlight the importance of the recovery between points and changeovers to recover and avoid overdue fatigue.

Hypohydration and dehydration could exacerbate the effects of repeated high-intensity efforts on the onset of NMF. Hornery et al., [26] found in amateur players that dehydration during a match from an international tournament was inversely correlated with serve tennis consistency (height of the tossing arm at ball release), which is a key component of serve velocity[27]. Their findings suggest a possible relationship between dehydration and NMF. On the other hand, in runner athletes, Maresh et al., [28]found a significant association between SalCC and hypohydration. The compiled evidence suggest a possible link between dehydration, increases in SalCC and NMF development. However, to our knowledge, this had not been fully investigated.

Therefore, the aim of this study was to measure if profesional male tennis players show an anticipated increase in SalCC before playing an official singles match played on clay-court, and develop NMF in association with increased cortisol levels after the match. Furthermore, we sought to investigate the associations between hydration status and NMF or cortisol levels. It is hypothesised that a singles profesional match would be associated to an anticipatory elevatation of salivary cortisol concentrations and the physiological and mechanical stress of the match would evidence neuromuscular fatigue.

## Methods

### Participants

Nine professional’s male tennis players, 4 seniors and 5 juniors (18.7 ± 1.8 years, 78.1 ± 8.2 kg of body mass; 1.82± 0.08 m of height, 11.8 ± 2.5% fat mass, 23.6 ± 1.8 kg/m^2^ of BMI) participated in this study. All participants were regularly competing in International Tennis Federation (ITF) events (Futures), training between 12–20 hours/week of technical/tactical in court tennis training and 5–10 hours/week of physical training. Three of the participants were professional tennis ranked between 1400–1800 Association of Tennis Professionals (ATP) and six were among the best 200 Spanish senior tennis players. Participants and parents of participants under 18 years old, were informed of all experimental procedures and a signed informed consent was collected from each participant. For the minors (<18) included in the study, consent from parents was obtained. The University of Murcia Bioethics Commission approved the study, which complied with the Declaration of Helsinki’s recommendations.

### Experimental design

**Fig 1:**
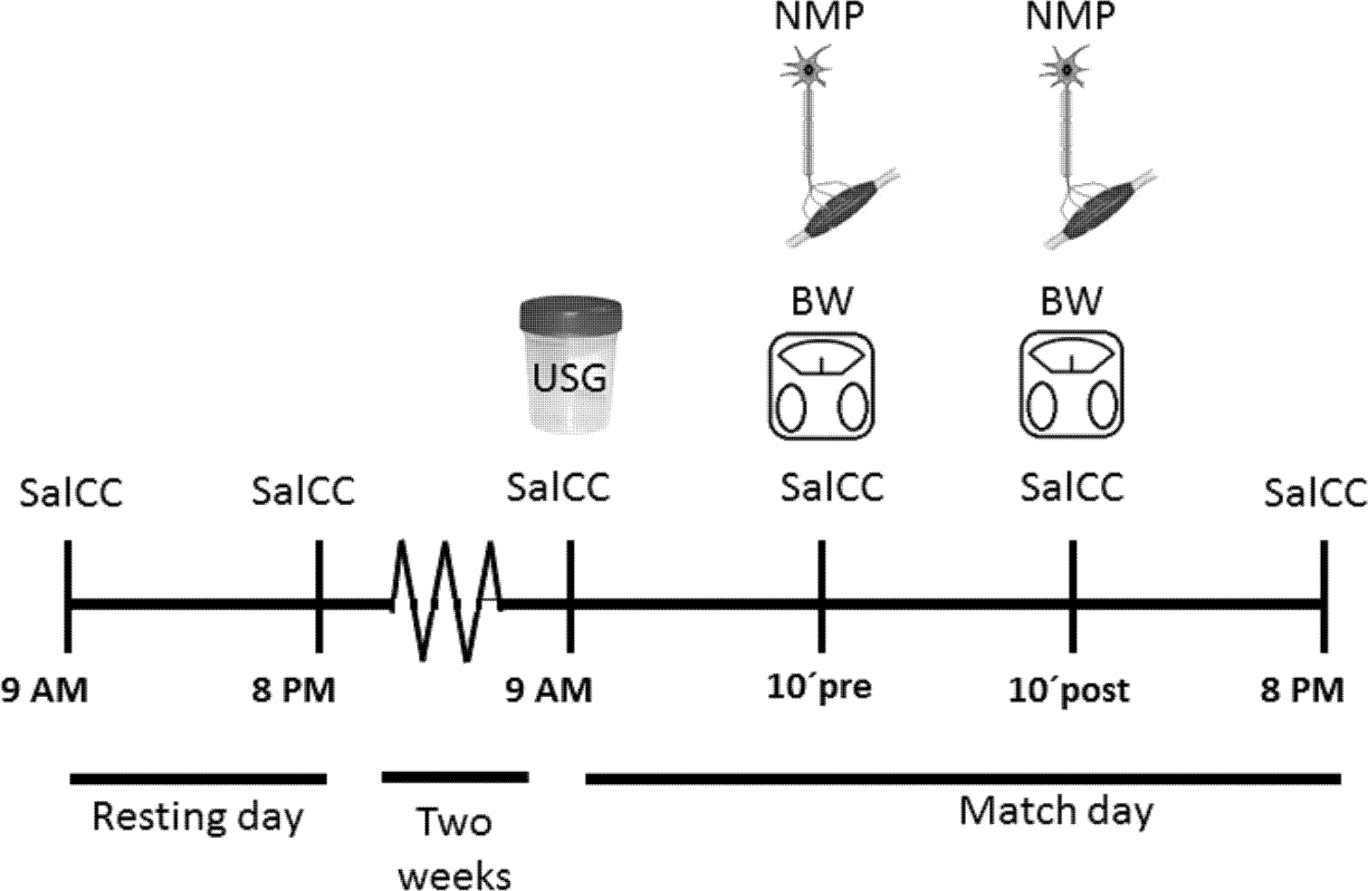
Experimental design.

A professional tennis tournament played in clay-court was selected to measure all the variables of interest. Recruited participants were tested 2 weeks before the first match of the tournament (rest day) and at the day of the first match of competition. Two salivary samples (9 am and 8 pm) were collected during the rest day and at the same hours on the day of the match. In addition, 10 minutes before and after the match, saliva samples were also collected. Finally, during the match day, hydration status, fluid turnover and neuromuscular tests were measured 10 min before and 10 min after the match (Fig 1).

### Testing protocol

Two weeks before the tournament, saliva samples were obtained at (9:00 am and 8:00 pm) following a previously described protocol [29]. Saliva samples were frozen at -20°C and stored until analysis. Urine samples were also collected to analyze on-site specific gravity with a pre-calibrated hand-held refractometer (URC-N_e,_ Atago, Japan) as previously described[30]. During the match day, after saliva and urine collection, participant’s body weight (Tanita B-601, Tokyo, Japan) and height (Seca 202, Hamburg, Germany) were measured. In addition, before and after the match, neuromuscular tests were conducted.

### Salivary cortisol concentrations

Two saliva samples were obtained on a resting day (24 hours without training) two weeks before tournament (C_1_ and C_2_) (9:00 am and 8:00 pm respectively) and 4 saliva samples were collected on the match day (9:00 am (C_3_), 10 minutes before the match (C_4_), 10 minutes after the match (C_5_) and in the evening at 8:00 pm (C_6_). Subjects chewed the cotton inside the Salivette tube for 60 seconds and then stored the sample (Sarstedt, Germany). Volunteers were instructed to collect the samples before eating (at least 8 hours fasting) and to thoroughly rinse their mouth with tap water before sampling to avoid saliva contamination. Also, to prevent saliva contamination caused by microinjuries [17] participants were required not to brush their teeth before the saliva collection. After collection, salivary samples were stored frozen at -20°C until analysis, following the general guidelines for saliva samples preservation[31]. Salivary cortisol was determined in duplicate using an enzyme immunoassay kit (Salimetrics Salivary Cortisol Kit; State College, PA) with a sensitivity of 0,007 μg/dl an average intra and inter-assay CVs of 5.5% and 4.9%, respectively. All the salivary samples of one individual were analyzed on the batch to reduce variability.

### Neuromuscular tests

Following the protocol used in previously published studies of neuromuscular performance [32], a standardized warm-up (i.e., 10 min of jogging and 10 min of joint mobilization exercises) was performed before testing. After the warm-up, participants started the neuromuscular test battery assessments under a strict paced schedule. Participants completed two repetitions of CMJ using an infrared photocell jump system (Optojump, Microgate, Italy) according to the standardized methodology described by Bosco et al.[33]. Each participant performed two maximal jumps with one minute of rest between trials. If jump height difference between trials was less than 5%, then average height of the two attempts was registered[34], otherwise, a third repetition was performed and the two closest averaged. Test-retest intra-class coefficient (ICC) and coefficient of variation (CV) were 1.0% and 3.2%, respectively. Then, volunteers stood at 0° degrees of elbow flexion and the forearm and elbow in a neutral position holding a calibrated handgrip dynamometer (Takei 5101, Tokyo, Japan) with the dominant hand. Participants performed two handgrip maximum isometric voluntary contractions with one minute of rest between trials. Mean value out of two attempts was recorded as the maximum voluntary handgrip strength. Additional attempts were performed until two consecutive measures differed less than 5%. Test-retest ICC and CV of HIS were 1.0% and 4.1%, respectively.

### Environmental conditions

Tennis matches were played in clay-courts according to the guidelines of the ITF. During the duration of the tennis matches, air temperature and humidity were monitored using a portable weather station (WMR 108, Mextech, India). Data were averaged to obtain the mean temperature (°C) and relative humidity (%).

### Statistical analysis

Shapiro Wilk-Test showed a normal distribution of data. All variables measured pre-and post-match were compared using student T-test for related samples. SalCC comparison between rest and match day was analyzed by repeated measures ANOVA, using hour of day (AM vs. PM) and condition (Rest vs. Match) as intra-subject factors. After a significant *F*-test differences among means were calculated with Bonferroni´s adjustment. Effect size (ES) was estimated and the results were based on the following criteria: trivial (0–0.19), small (0.20–0.49), medium (0.50–0.79) and large (≥0.80)[35]. Data from changes in pre-post-match values of SalCC, neuromuscular test battery, and percent body weight losses were analyzed using Pearson’s coefficient of correlation. Data are presented as mean and standard error of the mean (SEM); and the level of significance was set at P≤0.05. All the statistical analysis was done using SPSS software version 18 (SPSS Inc, Chicago, IL; USA).

## Results

### SalCC

**Fig 2:**
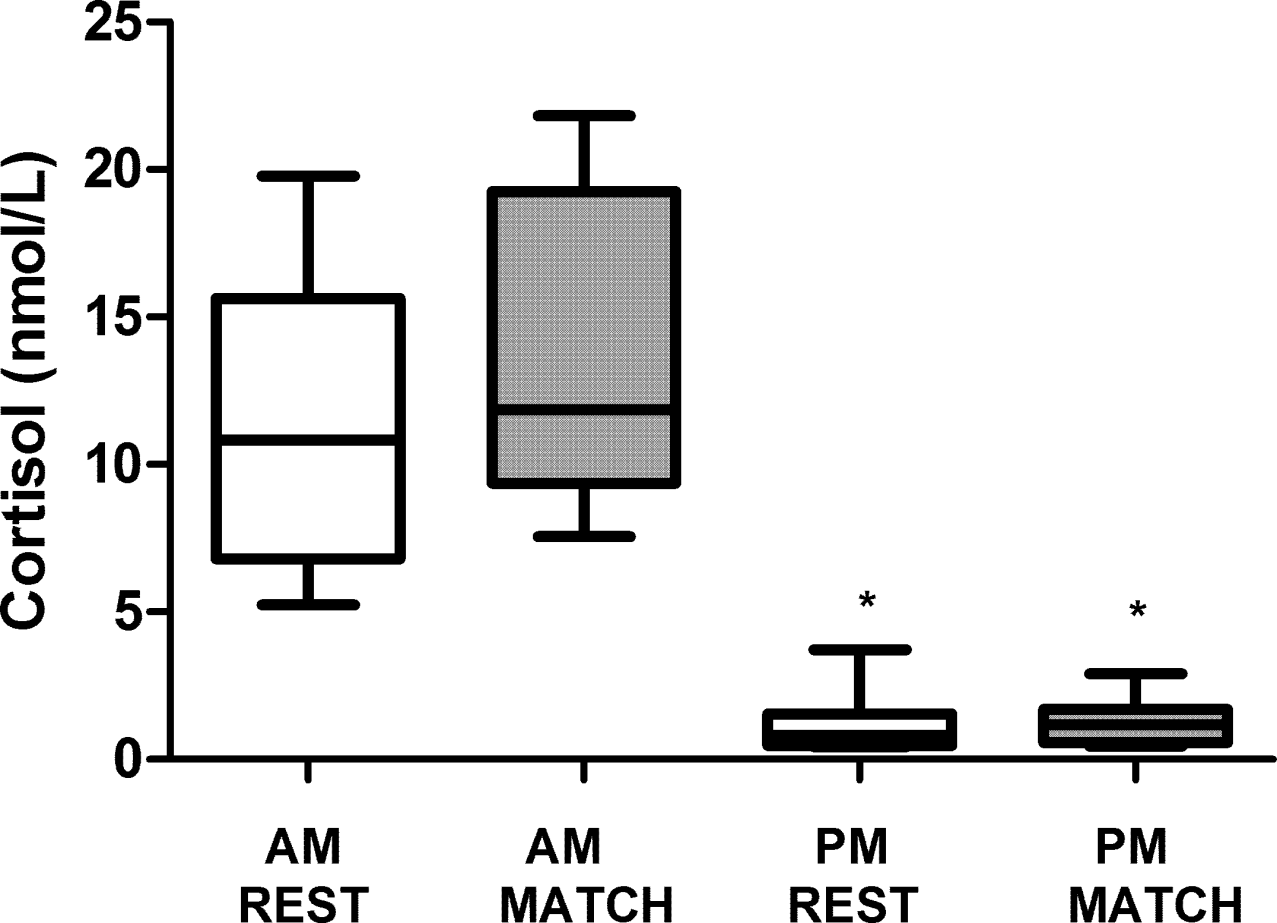
Comparison of Salivary cortisol concentrations (SalCC) between rest day (REST) and match day (MATCH). * Different from AM (9AM) (P<0.05).

**Fig 3:**
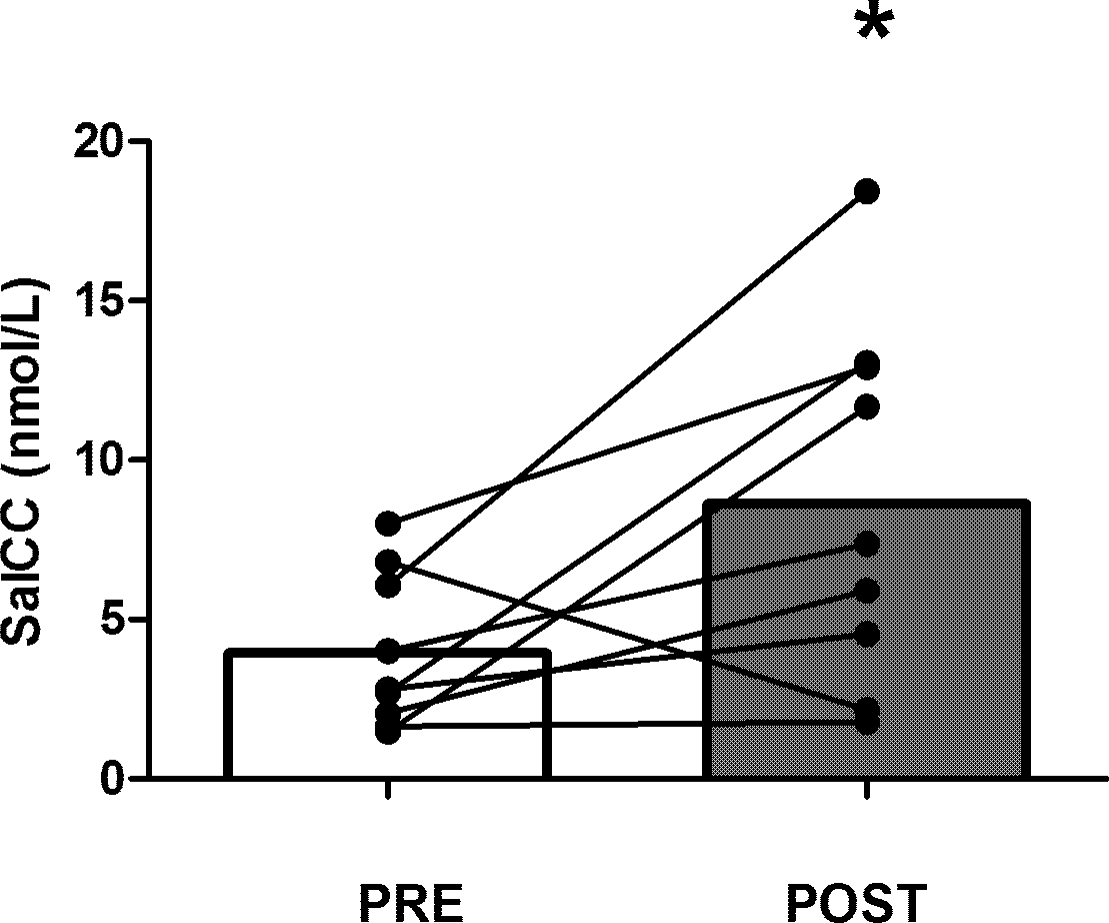
Salivary cortisol concentration (SalCC) 10 minutes before (PRE) and 10 minutes after (POST) a singles tennis match. Bars are means. Individual responses are displayed as dots linked with lines. * Denotes difference between PRE and POST measurements (P = 0.033).

SalCC differences between the rest and match day are displayed in Fig 2. In both, the rest and match day, SalCC was higher in the morning than in the evening (hour of day effect P < 0.001). However, neither in the morning (9:00 am) or in the evening (8:00 pm), SalCC was different between rest and match day (situation effect P = 0.161). Differences of SalCC 10 min pre-and post-match are depicted in Fig 3. SalCC increased a 2.2-fold from 10 minutes pre-Match 4.0 ± 2.4 nmol·L^-1^ to 10 minutes post-Match 8.7 ± 5.7 nmol·L ^-1^ (P = 0.033). Individual analysis of SalCC changes during the match showed that eight of 9 participants increased the SalCC after competition.

### Neuromuscular fatigue (CMJ and HS)

**Fig 4:**
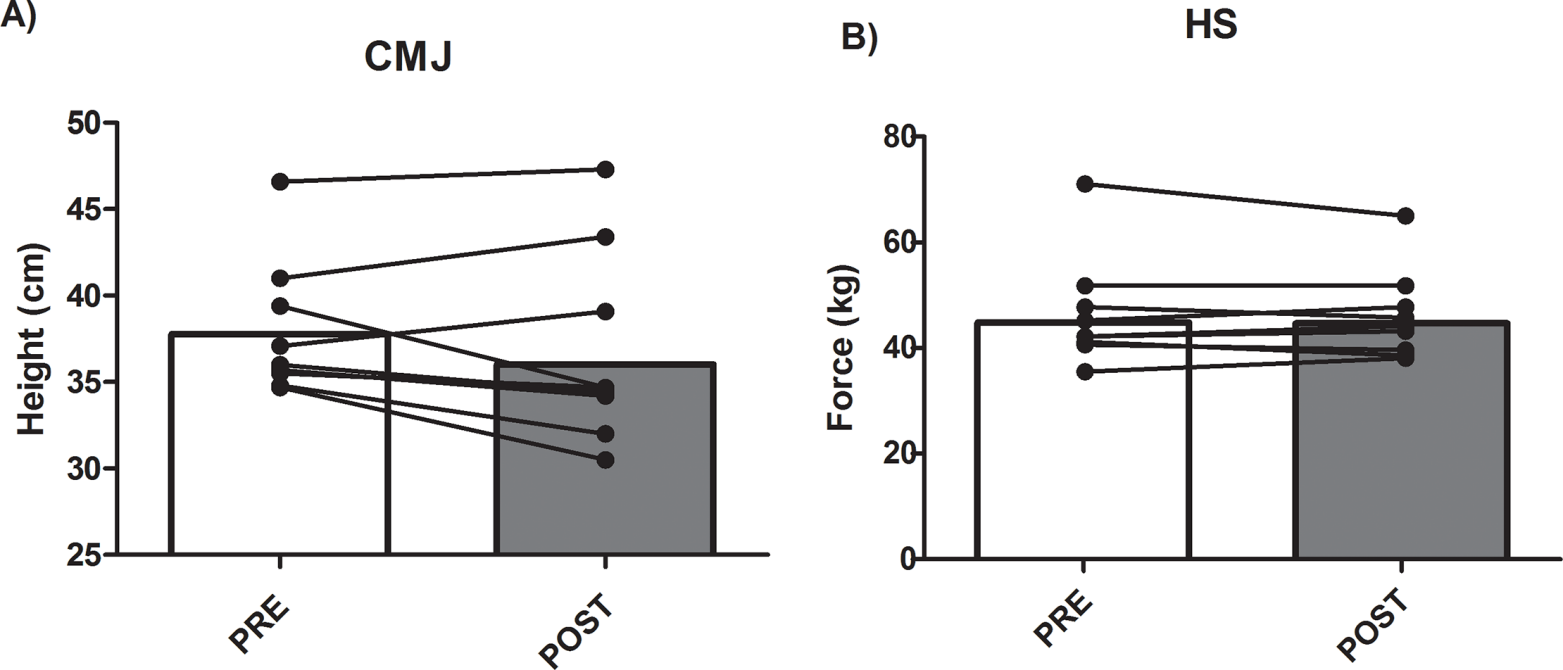
Neuromuscular performance before and after a professional tennis match. Bars are means. Individual responses are displayed as points linked with lines. CMJ, counter-movement jump; HS; handgrip isometric strength.

Results of CMJ and HS before and after the match are displayed in Fig 4. Although CMJ and HS decreased after the match (from 36.78 ± 1.50 cm to 35.49 ± 1.90 and from 44.81 ± 10.19 cm to 44.68 ± 8.29 kg, respectively), the observed decrement did not reach statistical significance in any of the two variables (P = 0.210, ES = 0.10 and P = 0.690, ES < 0.01, respectively). Individual analysis showed that six and four of nine participants reduced jump height and isometric handgrip force, respectively.

### Hydration status (USG, % dehydration and fluid ingestion rate)

**Fig 5:**
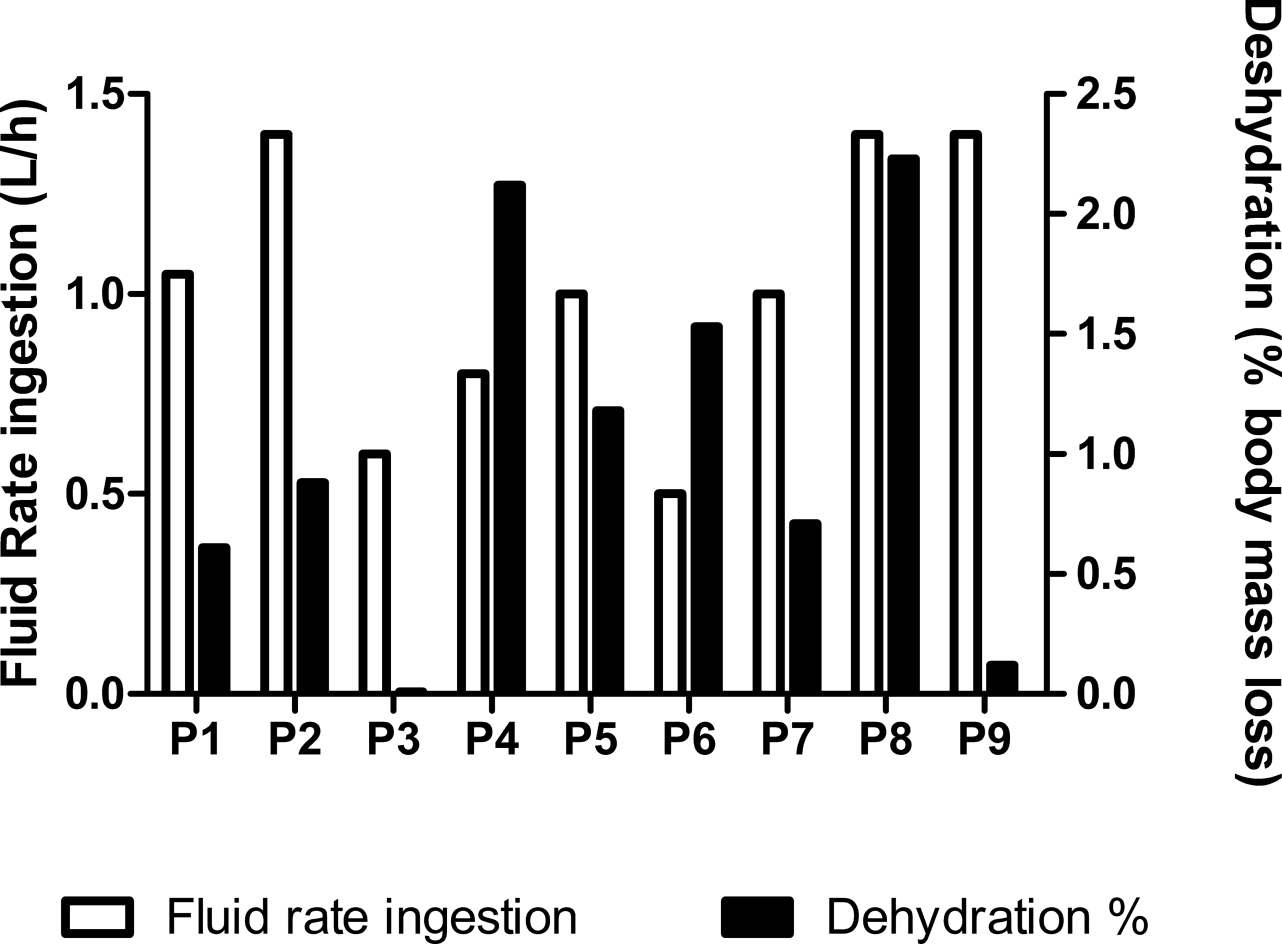
Individual response of fluid balance (L/h) and body weight lost (%) after a singles professional tennis match.

USG values indicated that as an average, participants arrived in hypohydrated state to the match (USG = 1.026 ± 0.002). Individual data of fluid rate ingestion and percentage of body mass loss during the match are displayed in Fig 5. Body mass decreased from 76.7 ± 2.8 kg to 75.9 ± 2.7 kg after the match (-1.0 ± 0.3%, P = 0.001, ES = 0.25), while fluid rate ingestion was 1.034 ± 0.124 L/h.

### Environmental conditions during match day

During the tennis match day, averages of air temperature and relative humidity were 29.7 ± 1.8°C and 48.1 ± 3.7%, respectively.

### Correlations

Pearson correlation coefficient between changes in the studied variables before and after the match showed that SalCC changes correlated significantly with body mass loss (r = -0.632; P = 0.033) and match duration (r = 0.802, P = 0.004). On the other hand, SalCC changes did not correlate significantly with CMJ changes (r = -0.513; P = 0.078) or HS changes (r = -0.212; P = 0.290). Finally, neither body mass loss nor match duration correlated with CMJ, (r = 0.577; P = 0.052 and r = -0.095; P = 0.403, respectively) or HS (r = -0.272; P = 0.239 and r = 0.210; P = 0.029, respectively).

## Discussion

The main finding of this study is that SalCC in the morning before an official match and the corresponding night, were not different from the concentrations measured at the same time points during a control-resting day, despite an acute increase in SalCC evidenced 10 minutes after the match. This suggest no anticipatory response to the game in the morning and fast recovery at night in these professional players. In addition, we show that the acute increase in SalCC occur without evidence of alteration of the mechanisms involved in muscle activation and in consequence force production (i.e. neuromuscular fatigue) in both, upper and lower extremities (CMJ and HS, respectively). It is remarkable that neuromuscular fatigue is not evident, despite participants competed in a hypohydrated state (USG>1.020) and during the match developed a mild dehydration (~ 1% body weight loss). Nonetheless, dehydration correlates with the increases in SalCC (r = -0.632; P = 0.034), suggesting that dehydration during a real game could increase the level of stress in professional tennis players.

In amateur tennis players, it has been documented an increase in SalCC concentrations in the hours before the match of a regional tournament compared to baseline levels[8]. In the present study, we did not find evidence of a significant increase in SalCC (17%, P = 0.161) in the morning before the match in comparison to SalCC measured during a resting day. Compared to the resting day, Filaire et al. [8]found that tennis players who lost a match showed higher SalCC (94% increase) the morning before match than those players who have won their matches (35% increase). Similar results have been observed in other studies conducted in other sports disciplines (e.g. soccer, judo and rugby;[14, 36, 37]). However, presently SalCC was not increased the morning before the match. This is similar to the findings reported by Elloumi et al., in professional rugby players [38] who participated in international and highly competitive tournaments. Thus, it is possible that the differences observed between amateur (Filarire and co-workers data) and professional players (present and Elloumi et al., studies) may be attributed to an increased tolerance to physiological stress in the latter. Unfortunately, we could not find a publication comparing the cortisol concentrations the day of an official match vs. a resting day in amateur vs. professional athletes. Therefore, this topic requires further investigation.

Cortisol concentrations is a well-recognized physiological stress marker[17]. This steroid hormone plays an important role in response to stress because of the activation of the hypothalamic-pituitary-adrenocortical axis. Our data suggest an acute increase of physiological stress immediately after the tennis match judging by the increase in SalCC. This finding is in agreement with other studies conducted in regional tennis players and elite soccer players[8, 39]. In contrast, professional soccer players reported minimal differences in SalCC after a training match [40] which seems to suggest the necessity of a real competition challenge to stimulate an increase in SalCC. Thus, SalCC may be considered as a useful tool to measure physiological stress in particular during competitive situations.

Data from high-level runners revealed a significant correlation between the acute increases in cortisol concentrations and NMF measured by CMJ (r = -0.782, P > 0.05;[29]). Since previous studies have demonstrated that CMJ is a feasible test for measuring neuromuscular fatigue[4], it was presently used to detect NMF, together with changes in salivary cortisol concentration. However, we did not find statistical differences between physiological stress and CMJ (r = -0.513, P = 0.078). These inconsistent findings, could be attributed to the different physiological demands between endurance and intermittent sports, since in a tennis match there are resting periods which may be long enough to prevent neuromuscular fatigue[25].

Dehydration in the range of 2% of body weight loss has been reported to have deleterious effects for endurance [41] and team sport performance[42]. According to our data, despite of the hypohydrated status of the players before the match and the mild dehydration (1.0% of body weight loss) induced by the tennis match, professional players did not show NMF. In contrast, it has been demonstrated that a mild dehydration (<1% body weight loss) is linked to reductions in neuromuscular performance (e.g. sprinting times) in tennis players[43]. Nonetheless, the results reported by Periard et al. study may be explained by extreme environmental conditions in which the tennis match was performed (37°C and 33% of relative humidity in comparison of 29.7± 1.8°C and 48.1 ± 3.7 RH in our study).

The present investigation has a main limitation, which is the reduced sample size (n = 9). However, it is worth to mention that recruiting professional tennis players during official competition is very difficult since not only the participants, also the tournament authorities should be coordinated to cooperate with the research during the competition. We hope that the fact of presenting for the first time, the evolution of the studied variables during a professional tennis tournament might mitigate the reduced sample size.

The present investigation could be summarized into three findings. Professional tennis players do not show an anticipatory stress response (SalCC) the morning in which they take part in a tennis professional match comparing to a rest day. This findings suggest that professional players may regulate better the physiological stress caused by a competitive tennis match compared to amateur players[8]. Secondly, the physiological and mechanical demands of the match are associated with increases in SalCC. Lastly, the variables measured did not provide evidence of NMF after the match, but the dehydration induced by the match correlated with the elevations in SalCC after the match suggesting that dehydration may augment the stress of a professional tennis match and that it should be avoided.

## Supporting information

S1 File Experimental data tennis study. (XLSX)
